# 295. Home Decolonization to Decrease UTI, Graft Failure, and Death After Renal Transplantation (PROTEKT: PROTEction after Kidney Transplant): A Pragmatic Quality Improvement Study

**DOI:** 10.1093/ofid/ofaf695.097

**Published:** 2026-01-11

**Authors:** Hannah H Nam, Uttam Reddy, Anna Escudero, Francis Baltazar, Thomas T Tjoa, Kathleen A Quan, Keith M Madey, Mariya Kovryga-Kornick, Susan Huang

**Affiliations:** University of Irvine - California, Orange, CA; University of California - Irvine, Orange, California; University of California - Irvine, Orange, California; University of California - Irvine, Orange, California; University of California, Irvine School of Medicine, Division of Infectious Diseases, Irvine, California; University of California Irvine Health, Orange, California; University of California - Irvine, Orange, California; University of California - Irvine, Orange, California; University of California, Irvine School of Medicine, Irvine, California

## Abstract

**Background:**

Infections are a major cause of morbidity and mortality in kidney transplant recipients. Urinary tract infections (UTI) are common early complications associated with graft loss, hospitalization, and death. We sought to evaluate whether post-transplant home decolonization could reduce complications within 6 months of transplant.

**Figure:**
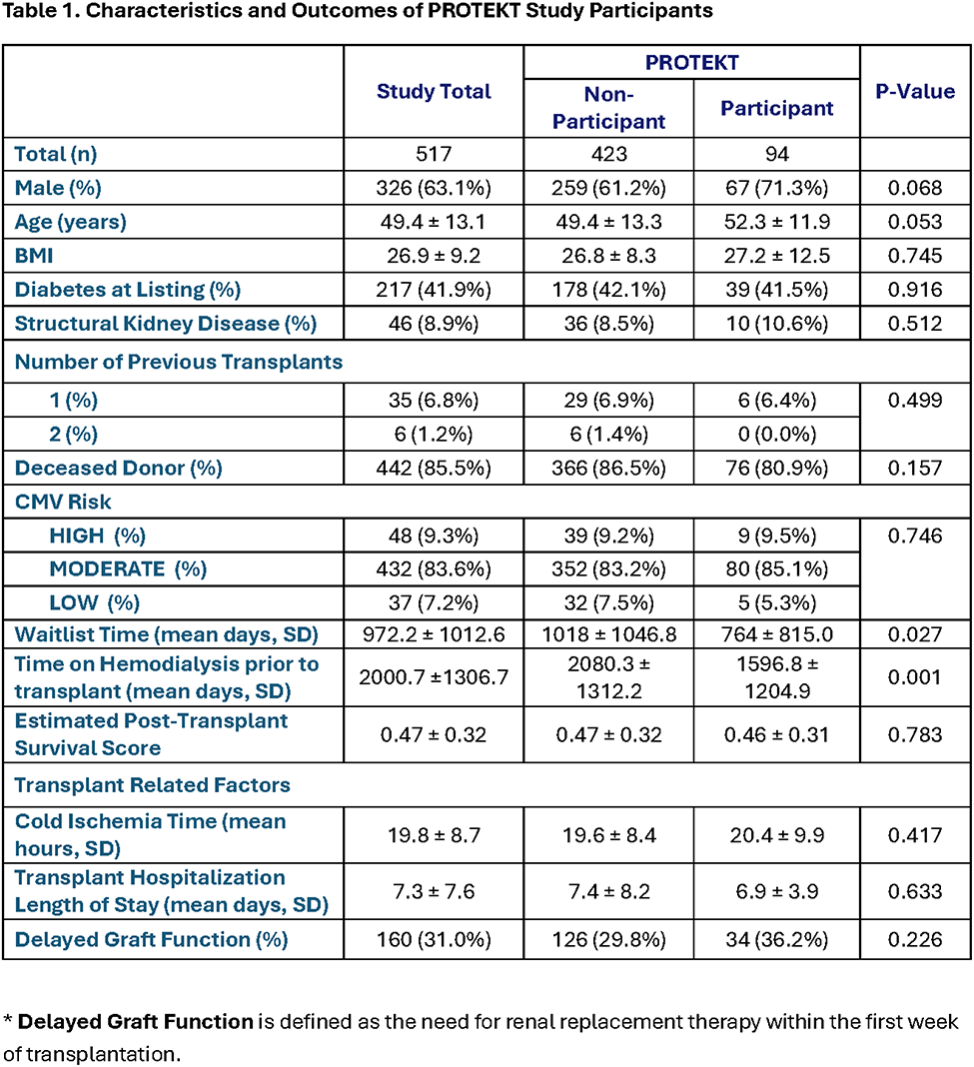

**Figure:**
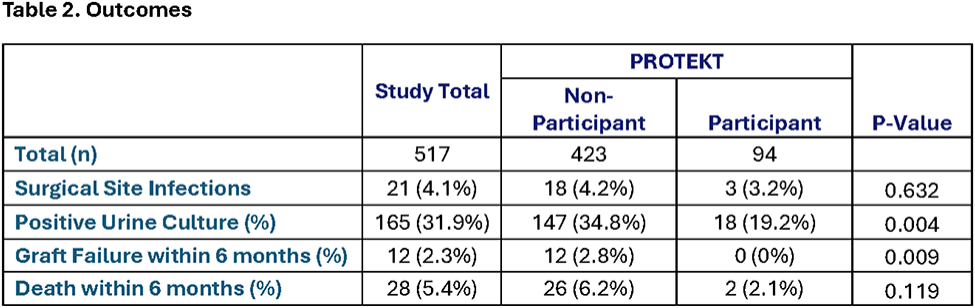

**Methods:**

We conducted a pragmatic pre-post quasi-experimental study of adult kidney transplant recipients whereby a convenience sample of transplant recipients received post-discharge decolonization during a 16-month intervention period (Feb 1, 2023 to May 30, 2024). Post-discharge kits included an instructional booklet and 2% CHG cloths for daily cleansing of the surgical incision site and perineum for 3 months following transplantation. Patients were provided their first kit and instructions by transplant nurses. Subsequent monthly kits were mailed to the patient’s home.

Participants were compared to non-participants during the intervention period and from a 2-year pre-intervention period (Jan 1, 2021 to Jan 30, 2023). Outcomes included time to bacteriuria ( >100K CFU/ml) within 3 months of transplant, and graft failure or death within 6 months of transplant. Outcomes were assessed using Kaplan-Meier survival analysis and Cox proportional hazards models adjusting for baseline differences between groups.

**Figure:**
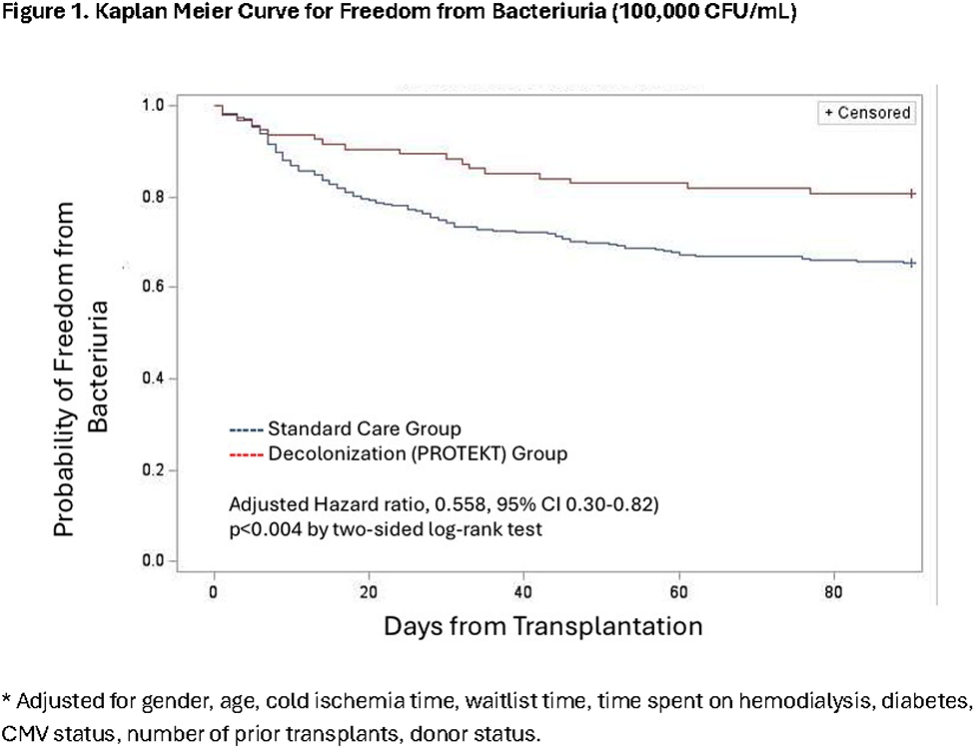

**Results:**

Characteristics of the 517 patients (94 participants, 423 non-participants) are found in [Table 1]. Recipients of the decolonization intervention had significantly fewer UTI (34.8% vs 19.2%) and graft failure (0% vs 2.8%) events compared to non-participants (p < 0.05). Deaths were reduced, but not statistically significant (2.1% vs 6.2%) [Table 2]. K-M curves confirmed higher bacteriuria-free survival than controls at 30, 60, and 90 days (88.3% vs. 74.5%; 83.0% vs 67.1%, and 80.9% vs 65.3%, respectively; log-rank p< 0.004), and adjusted analyses showed a significantly lower risk of UTI (aHR 0.56, 95% CI 0.30-0.82; p< 0.004) [Figure 1]. Adverse events were rare (∼1%).

**Conclusion:**

Post-discharge CHG bathing was associated with significantly reduced UTI, graft failure and death in kidney transplant recipients. This safe, simple, and inexpensive strategy may offer a novel, resistance-sparing alternative to antibiotics.

**Disclosures:**

All Authors: No reported disclosures

